# 

**Published:** 1891-05-16

**Authors:** 


					INBO
for INFANTS
' ISINGLASS
AND INVALIDS.
No. 242. Vol. X.]
Saturday,
May 16th, 1891 .*3
[One Pbnny.

				

